# Of Older Mice and Men: Branched-Chain Amino Acids and Body Composition

**DOI:** 10.3390/nu11081882

**Published:** 2019-08-13

**Authors:** Rosilene V. Ribeiro, Samantha M. Solon-Biet, Tamara Pulpitel, Alistair M. Senior, Victoria C. Cogger, Ximonie Clark, John O’Sullivan, Yen Chin Koay, Vasant Hirani, Fiona M. Blyth, Markus J. Seibel, Louise M. Waite, Vasi Naganathan, Robert G. Cumming, David J. Handelsman, Stephen J. Simpson, David G Le Couteur

**Affiliations:** 1 School of Life and Environmental Sciences, Faculty of Science, The University of Sydney, Sydney 2006, Australia; 2 Charles Perkins Centre, Camperdown, The University of Sydney, Sydney 2006, Australia; 3 Sydney Medical School, Faculty of Health and Medicine, The University of Sydney, Sydney 2006, Australia; 4 Heart Research Institute, The University of Sydney, Sydney 2006, Australia; 5 ARC Centre of Excellence in Population Ageing Research (CEPAR), Kensington 2033, Australia; 6 Concord Clinical School, Faculty of Health and Medicine, The University of Sydney, Concord 2139, Australia; 7 ANZAC Research Institute, The University of Sydney, Concord 2139, Australia; 8 School of Public Health, University of Sydney, Sydney 2006, Australia; 9 Ageing and Alzheimers Institute, Concord Hospital, University of Sydney, Concord 2139, Australia

**Keywords:** branched-chain amino acids, body composition, ageing, mice, humans

## Abstract

Protein and branched-chain amino acid (BCAA) intake are associated with changes in circulating BCAAs and influence metabolic health in humans and rodents. However, the relationship between BCAAs and body composition in both species is unclear, with many studies questioning the translatability of preclinical findings to humans. Here, we assessed and directly compared the relationship between circulating BCAAs, body composition, and intake in older mice and men. Body weight and body fat were positively associated with circulating BCAA levels in both mouse and human, which remained significant after adjustments for age, physical activity, number of morbidities, smoking status, and source of income in the human cohort. Macronutrient intakes were similarly associated with circulating BCAA levels; however, the relationship between protein intake and BCAAs were more pronounced in the mice. These findings indicate that the relationship between circulating BCAAs, body composition, and intakes are comparable in both species, suggesting that the mouse is an effective model for examining the effects of BCAAs on body composition in older humans.

## 1. Introduction

Branched-chain amino acids (BCAAs; valine, leucine, isoleucine) have been linked to the development of type 2 diabetes, insulin resistance, and obesity in both rodents and humans [1,2,3,4,5]. It is unclear whether this association is a direct reflection of the diet, or the consequence of systemic metabolic derangement [3,6,7]. In mice, increasing intake of proteins and BCAAs is associated with both elevated circulating BCAA levels and changes in body composition [3,4,6,8]. In humans, however, the link between dietary BCAAs and health is less clear. While some studies linking high circulating levels of BCAAs with metabolic dysregulation also show increases in total protein and/or BCAA intake [1,2,9,10,11], other observational studies report conflicting evidence, with some showing positive, negative, or neutral effects of dietary protein and/or BCAAs on health [12,13].

Therefore, the question of how closely the relationship between diet, BCAAs, and body composition in rodents reflect the relationship in humans is an important issue to resolve. In animal studies, elucidating the relationship between diet, circulating BCAAs, and body composition can be uniquely informative, controlling for various factors that could not otherwise be achieved in a free-living human population. However, translating these findings in order to humans to better understand the role of BCAAs in health requires that key outcomes in both rodents and humans respond similarly.

Here, we directly compare the relationship between circulating BCAAs, energy and macronutrient intake, and body composition in a group of men aged over 75 years with a cohort of older mice at 15 months of age. We show that the relationship between circulating BCAAs, intake, and body composition were comparable across both free-living humans and mice under tightly controlled dietary manipulations, suggesting that preclinical findings in mice may reliably inform studies examining the link between BCAAs and body composition in humans.

## 2. Materials and Methods

### 2.1. Human Cohort

#### 2.1.1. Study Design

The Concord Health and Ageing in Men Project (CHAMP) is a longitudinal cohort study investigating the health of older men based in Sydney, Australia. The recruitment, which is described elsewhere [14], involved a defined urban region (the Local Government Areas of Burwood, Canada Bay, and Strathfield near Concord Hospital in Sydney, Australia) sampled using the New South Wales Electoral Roll. A total of 1705 men participated in the initial study wave in 2005–2006 [14]. Dietary data were assessed through diet history interviews at the third wave (five-year follow-up, 2012). At the five-year follow-up, 954 participants completed questionnaires and clinical assessments, and of those, 794 completed diet history interviews. CHAMP was approved by the Sydney South West Area Health Service Human Ethics Committee, Concord Repatriation General Hospital, Sydney, Australia (Protocol number HREC/10/CRGH/28). Written consent was obtained from all participants and the study was conducted in accordance with the World Medical Association Helsinki Declaration.

#### 2.1.2. Dietary Data

Details about CHAMP dietary data collection and validation have been previously described [15]. Briefly, usual dietary intake (past 3 months) was assessed through diet history interviews [16]; those interviews followed a standardised protocol and were conducted at participants’ residences by a trained dietitian. Validation of this method was obtained through comparison with a 4-day weighed food record collected in a subgroup (*n* = 56) of participants [17].

#### 2.1.3. Outcome Variables

Body weight was measured according to a standardised protocol. All fasting blood tests were performed at the Diagnostic Pathology Unit of Concord RG Hospital, which is a National Australian Testing Authority (NATA) accredited pathology service, using a MODULAR Analytics system (Roche Diagnostics, Castle Hill, Australia). Amino acids were analysed at the Australian Proteome Analysis Facility, Macquarie University, using the Waters AccQ-Tag Ultra Chemistry Kit (Waters Corporation, Milford, MA, USA). Dual X-ray absorptiometry (DXA) using the fan beam Discovery-W scanner (Hologic Inc., Bedford, MA, USA) was used to obtain individuals’ whole-body scans. Men removed jewellery and wore a light cotton gown free from metal. The head was excluded from all measures and lean mass was calculated as the difference between non-fat mass and bone mineral content.

#### 2.1.4. Covariates

Information on socio-demographic, lifestyle, and economic factors were obtained through a self-completed questionnaire. Activity level was determined through the Physical Activity Scale for the Elderly (PASE) [18]. Source of income was categorised as means-tested age pension only and other (repatriation pension, veteran’s pension, superannuation or other private income, own business/farm/partnership, wage or salary, other or any source of income combination). Source of income was used as a proxy of personal income, assuming that age pensioners had the lowest income. Participants self-reported whether they have been diagnosed (by a doctor or a health care provider) with diabetes, thyroid problems, osteoporosis, Paget’s disease, stroke, Parkinson’s disease, kidney stones, dementia, depression, epilepsy, hypertension, myocardial infarction, angina, heart failure, intermittent claudication, chronic obstructive lung disease, liver disease, chronic kidney disease, arthritis, or cancer (excluding non-melanotic skin cancer and benign tumours such as bowel polyps and meningioma), and number of morbidities was calculated as the sum of diagnosed conditions.

### 2.2. Mouse Cohort

#### 2.2.1. Animals and Husbandry

A total of 384 C57BL/6J mice of both sexes were purchased from the Animal Resource Centre (Perth, WA) at 3 weeks old. Mice were group-housed in cages of four on a 12 h light–dark cycle at the Charles Perkins Centre at the University of Sydney. Mice were housed at 22–24 °C and were given ad libitum access to food and water. At 12 weeks of age, mice were randomly allocated to one of four experimental diets, monitored weekly, with food and body weights recorded bi-weekly until 6 months of age, and monthly thereafter. Animals remained on allocated diets throughout their lifetime. At 15 months of age, one mouse from each cage was sacrificed with tissues and blood collected for analysis (*n* = 12 males and 12 females). For comparisons between BCAAs and body composition, 19–23 mice per diet treatment were analysed. Data relating to cardiometabolic health and brain ageing have been published previously [19]. Experiments were approved by the University of Sydney’s Animal Ethics Committee (protocol no. 2014/752).

#### 2.2.2. Experimental Diets

Four experimental diets were designed and manufactured in dry-pelleted form by Specialty Feeds (Perth, Australia). Diets had the same energy density (14.4 kJ/g), were matched in net metabolizable energy from fat (18%), but varied in protein (P) and carbohydrate (C) content. Diets consisted of either 5% P and 77% C, 10% P and 72% C, 15% P and 67% C, or 19% P and 63% C. Diets were based on the rodent diet AIN-93G and formulated to contain all essential vitamins, minerals, and amino acids for growth in mice. Casein was the primary protein component, the main carbohydrate component was wheatstarch, and the main fat component was soy oil.

#### 2.2.3. Outcome Variables

Body composition and plasma amino acids were assessed in each mouse at 15 months of age. Body composition was assessed by EchoMRI (EchoMRI 900, Houston, TX, USA). Fat mass (g) and lean mass (g) were measured for each mouse and % body fat and % body lean calculated. Free amino acids were quantified in plasma. Targeted LC-QQQ-MS analysis was employed to detect metabolites. Metabolomic analysis was conducted using a tandem liquid chromatography–mass spectrometry (LC-MS/MS) system (Agilent 1260 Infinity liquid chromatography coupled to a QTRAP 5500 mass spectrometer (AB SCIEX, Framingham, USA). A hydrophilic interaction chromatography (HILIC) column was used for the detection of BCAAs. All raw data files (Analyst software, version 1.6.2; AB Sciex, Foster City, CA, USA) were imported into Multi-QuantTM 3.0 Software for MRM Q1/Q3 peak integration. A detailed description of metabolite profiling is described by O’Sullivan, et al. [6,20].

#### 2.2.4. Statistical Analyses

Data were analysed in R (v.3.4.1, R Foundation for Statistical Computing, Vienna, Austria) and significance considered when *p* < 0.05. Data from males and females were combined to analyse various outcomes in mice. Regression analyses were performed using the *lm* function in the base package [21,22]. Univariate analyses were performed to investigate the associations between circulating BCAAs, energy and macronutrient intake, and body composition variables. Two sample *t*-tests were performed to assess the mean difference between categorical variables (income and smoking status). Linear regressions were used to investigate the association between BCAA, age, physical activity level, and number of comorbidities. Subsequently, multivariate analyses were performed to adjust firstly for age and then for all factors commonly associated with dietary intake and circulating BCAA levels in humans. Correlation matrices were generated in R using the ”*corrplot*” package [23].

## 3. Results

The human cohort characteristics have been described elsewhere [15,23]. Briefly, the mean age was 81.3 years (SD = 4.6), body weight was 78.0 kg (SD = 12.78), fat mass 21.6 kg (SD = 7.22), percentage fat 29.5% (SD = 6.06), lean mass 47.8 kg (SD = 6.24), percentage lean 67.6% (SD = 5.90), and the majority of participants were non-smokers (96%), did not rely on the age pension as sole source of income (59%), and had at least two comorbidities. A total of 83 mice were analysed to determine the relationship of BCAAs with body composition. Mean body weight was 40.5 g (SD = 7.81), fat mass 14.0 g (SD = 5.59), percentage fat 33.4% (SD = 9.37), lean mass 24.1 g (SD = 3.74) and percentage lean 60.5% (SD = 8.79).

### 3.1. Circulating BCAAs and Body Composition

**Figure 1:**
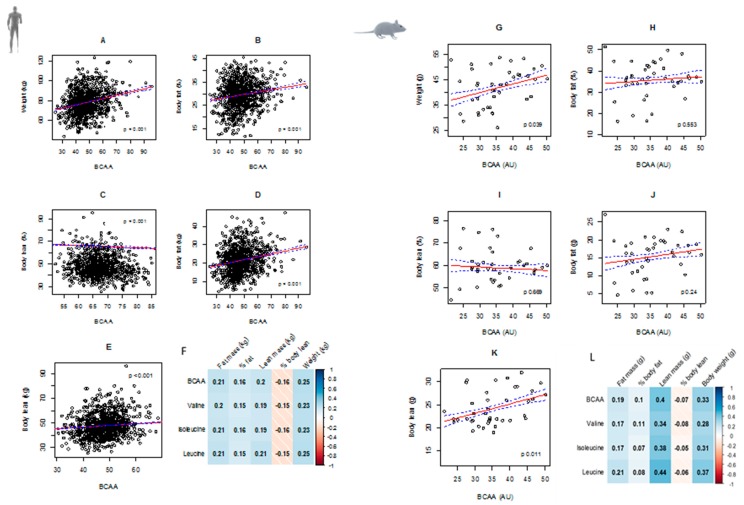
Circulating branched chain amino acids (BCAAs) and body composition in older men and mice. Red lines represent fitted values and blue dotted lines represent 95% confidence intervals. Body weight (kg), body fat (% and kg), and body lean (% and kg) were significantly associated with circulating BCAAs in men (**A**–**F**) and mice (**G**–**L**). Correlation matrices show similar results in both men (**F**) and mice (**L**): Energy, protein, and fat intake passively correlated with BCAAs whereas carbohydrate negatively correlated with BCAAs.

In humans, circulating BCAA levels significantly decreased with age (*p* < 0.001, Supplementary Materials, Figure S1). Body weight, fat, and lean mass as well as percentage body fat were positively associated with circulating BCAAs, whereas percentage body lean mass was negatively associated with BCAAs in both the human cohort and mice model, with stronger correlation in mice (Figure 1). After adjustment for age, physical activity, number of morbidities, smoking status, and source of income, the association between circulating BCAAs and all body composition measures remained statistically significant in the human cohort (Table 1). Individual BCAAs were also correlated with body composition variables in Figure 1F,L. We found that all outcomes, except for % body lean, were positively correlated with valine, isoleucine, and leucine—a result consistent in both mice and men.

### 3.2. Circulating BCAAs and Diet

**Figure 2:**
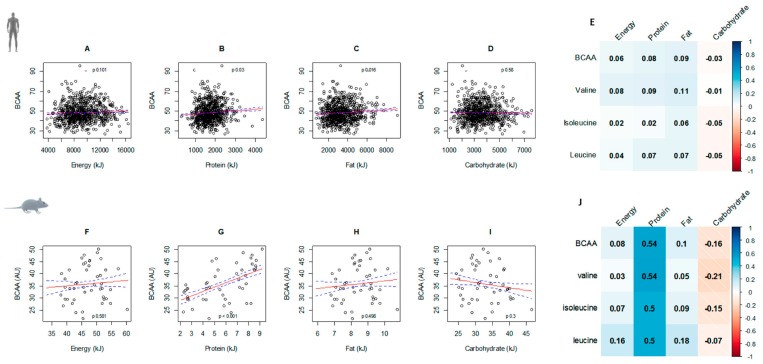
Circulating BCAA (branched chain amino acid) vs. nutrient intake in older men (**A–E**) and mice (**F–J**). Red lines represent fitted values and blue dotted lines represent 95% confidence intervals. In both humans and mice, protein intake was associated with higher circulating BCAAs (**B**,**G**). Men who consumed a diet high in fat were more likely to have higher circulating BCAAs (**C**). Correlation matrices show a similar pattern of correlation between dietary intakes and individual BCAAs in both men (**E**) and mice (**J**).

The association between energy, macronutrient intakes, and circulating BCAAs were similar in both mice and humans. Higher intakes of protein were significantly associated with higher circulating BCAAs and while similar trends occurred for energy intake, this was not statistically significant. Similarly, the negative relationship between BCAAs and carbohydrates showed a similar pattern in both species, with the correlation stronger in mice compared to humans. Fat intake was positively associated with circulating BCAAs in humans (*p* = 0.016), with a similar—albeit non-significant—trend in mice (Figure 2). Correlation matrices of individual BCAAs show similar relationships with energy and macronutrient intakes in both mice and men. With the exception of carbohydrate intake, all other nutrient intakes were positively correlated with valine, isoleucine, and leucine. As with total BCAA levels, individual amino acids were most strongly correlated with protein intake in mice, a result consistent with our previous findings [4].

## 4. Discussion

There has been limited research into the relationship between blood levels of BCAAs and aging. Studies have suggested that BCAAs decline with old age [24,25,26]. There have also been studies of the relationship between BCAAs and frailty or sarcopenia, which are classic age-related syndromes, some of which have shown an association with low BCAA levels [27,28,29,30]. Although it is unclear exactly why BCAAs decline with age, age-related changes in muscle protein metabolism, such as an imbalance in the rate of protein synthesis and breakdown, increased resistance to anabolic factors, changes in levels of physical activity, and an overall reduction in dietary intake of protein may be contributing factors [31,32]. Here, we demonstrate that total BCAA levels and individual levels of isoleucine, leucine, and valine, not only declined with age in humans, but were also linked with body composition, with those with higher circulating BCAA levels more likely to be carrying more weight and fat mass. Because the increase in fat mass outweighed the increase in lean mass, there was a corresponding increase in body fat percentage and a decrease in body lean percentage. Strikingly, we found that the association between BCAA and body composition markers were similar in mice models and in the human cohort of older males.

In terms of the associations between dietary intakes and circulating BCAA levels, as expected, a clear association was demonstrated between protein intake and circulating BCAA levels in mice. This association was not as clear in older men, though it followed the same trend. Plasma BCAA levels are likely to reflect long-term protein intake [33], yet high protein intake can increase circulating concentrations of BCAAs, with up to 80% of consumed BCAAs reaching circulation [34,35]. A number of factors, such as insulin resistance [36], cardiovascular disease [37], body composition [7], and protein sources [5], can also influence circulating BCAA levels. In the current study, we investigated the correlation between protein and BCAA levels in mice kept in a well-controlled environment for their whole life, whereas for the human cohort, dietary intake was estimated through diet history interviews, which may also explain the weaker association between protein intake and circulating BCAAs in the human cohort. Another potential explanation for the difference in the strength of association between protein intake and BCAA levels in mice and humans was the source of protein: mice were provided with casein protein, whereas protein sources were varied in the human cohort [15].

In recent years, BCAAs have received increased attention for their role in muscle synthesis and as a potential intervention option to improve muscle quality; a number of clinical studies have pointed to a crucial role of BCAAs in tackling muscle loss in older individuals. It has been suggested that the increase of protein or amino acids alone (as opposed to combining high protein or amino acids with high carbohydrate and/or energy) should result in better outcomes in older individuals [31,38]. The current study indicates that carbohydrate intakes have a negative association with circulating BCAA levels in both mice and humans.

The current study has several strengths worth mentioning: (1) data from a large sample of community-dwelling men and a large sample of mice on a well-defined diet and tightly controlled environmental conditions were used in our analyses; (2) the use of gold-standard methodology to obtain body-composition measures in humans (DXA) and in mice (EchoMRI); and (3) the dietary assessment method used (diet history) and frequent food intake measurements in mice. It is also worth mentioning the limitations of the current study: firstly, causal association cannot be established from the human cohort results; secondly, there may be other factors that play an important role in the relationship between dietary intake, BCAA levels, and body composition, such as genetic background and long-term dietary intake. Finally, the results may not be applicable to older females.

## 5. Conclusions

This is the first study to investigate the role of BCAA levels in large cohorts of mice and humans. Higher protein intakes were associated with higher BCAA levels and higher body weight and fat mass in both species. Further studies are required to elucidate the association between BCAA levels and body composition in older age, but this study adds to the body of evidence showing that mouse models can provide valuable insight into the relationship between nutrition and health, which can be translated to humans.

## Figures and Tables

**Table 1:** Regression coefficients (and SE) for change in body composition and macronutrient intake per unit of BCAA.

**HUMANS**		
**Fat mass (kg)**	**β (SE)**	***p***
Model 1	0.16 (0.02)	<0.001
Model 2	0.15 (0.03)	<0.001
Model 3	0.15 (0.03)	<0.001
**Body fat (%)**		
Model 1	0.10 (0.02)	<0.001
Model 2	0.10 (0.02)	<0.001
Model 3	0.10 (0.02)	<0.001
**Lean mass (kg)**		
Model 1	0.13 (0.02)	<0.001
Model 2	0.10 (0.02)	<0.001
Model 3	0.11 (0.02)	<0.001
**Body lean (%)**		
Model 1	−0.10 (0.02)	<0.001
Model 2	−0.10 (0.02)	<0.001
Model 3	−0.10 (0.02)	<0.001
**Weight (kg)**		
Model 1	0.33 (0.04)	<0.001
Model 2	0.28 (0.04)	<0.001
Model 3	0.30 (0.04)	<0.001
**MICE**		
**Fat mass (g)**	**B (SE)**	***p***
Model 1	0.14 (0.11)	0.24
**Body fat (%)**		
Model 1	0.11 (0.19)	0.55
**Lean mass (g)**		
Model 1	0.2 (0.07)	0.01
**Body lean (%)**		
Model 1	−0.07 (0.17)	0.67
**Weight (g)**		
Model 1	0.34 (0.16)	0.04

Model 1 was unadjusted (humans and mice). Model 2 was adjusted for age. Model 3 was adjusted for age plus physical activity, number of morbidities, smoking status, and source of income.

## Supplementary Materials

The following are available online at https://www.mdpi.com/2072-6643/11/8/1882/s1, Figure S1: Circulating BCAAs and age, PASE, number of morbidities, smoking status and source of income.
