# Supplementary material for: Of Older Mice and Men: Branched-Chain Amino Acids and Body Composition
Source: Nutrients. 2019 Aug 13;11(8):1882. doi: 10.3390/nu11081882 (PMC6723310; doi:10.3390/nu11081882)
Supplement: Supplementary file 1 [file nutrients-11-01882-s001.pdf]

Figure S1 – Circulating BCAAs and age, PASE, number of morbidities, smoking status and source of income

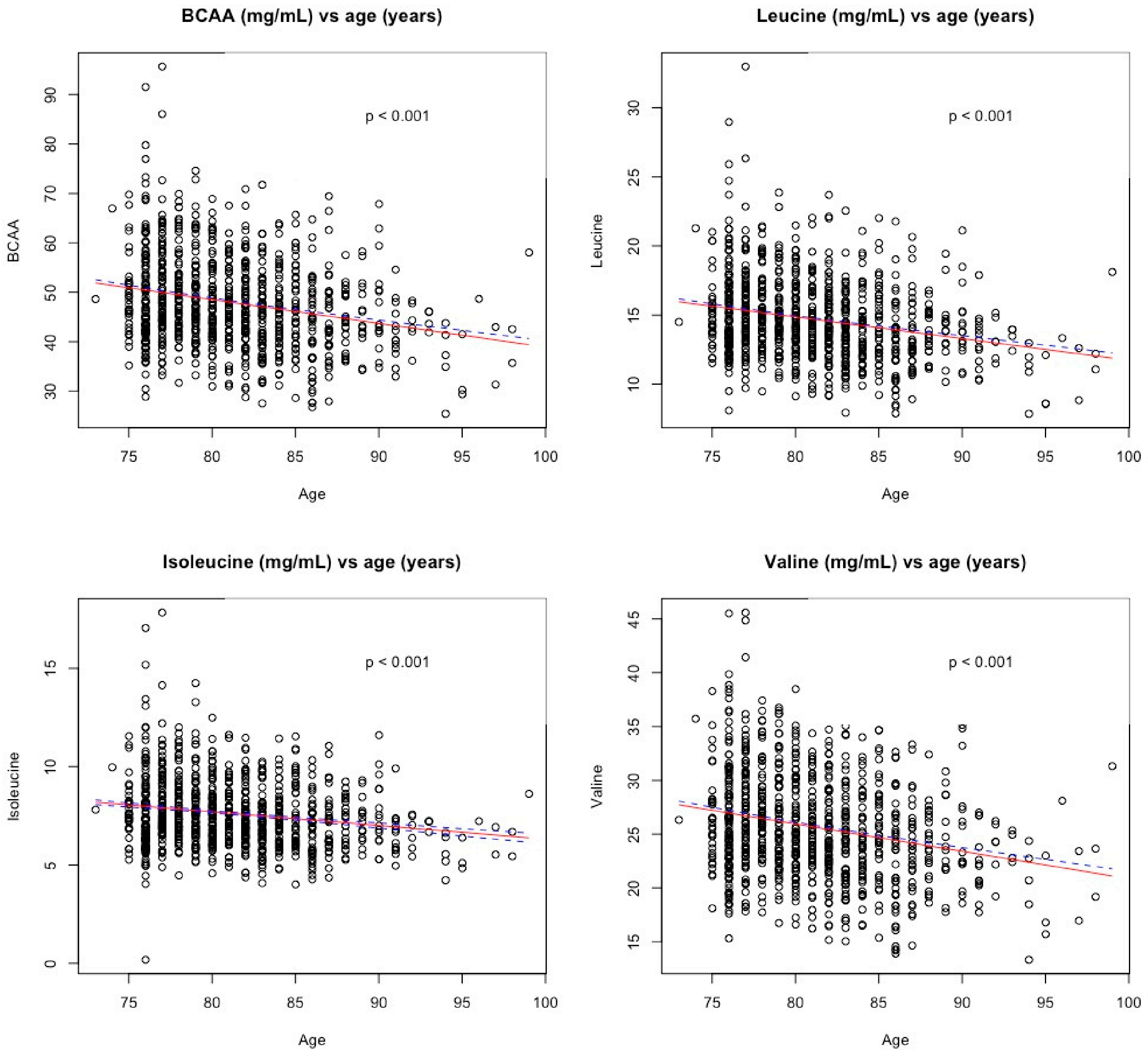

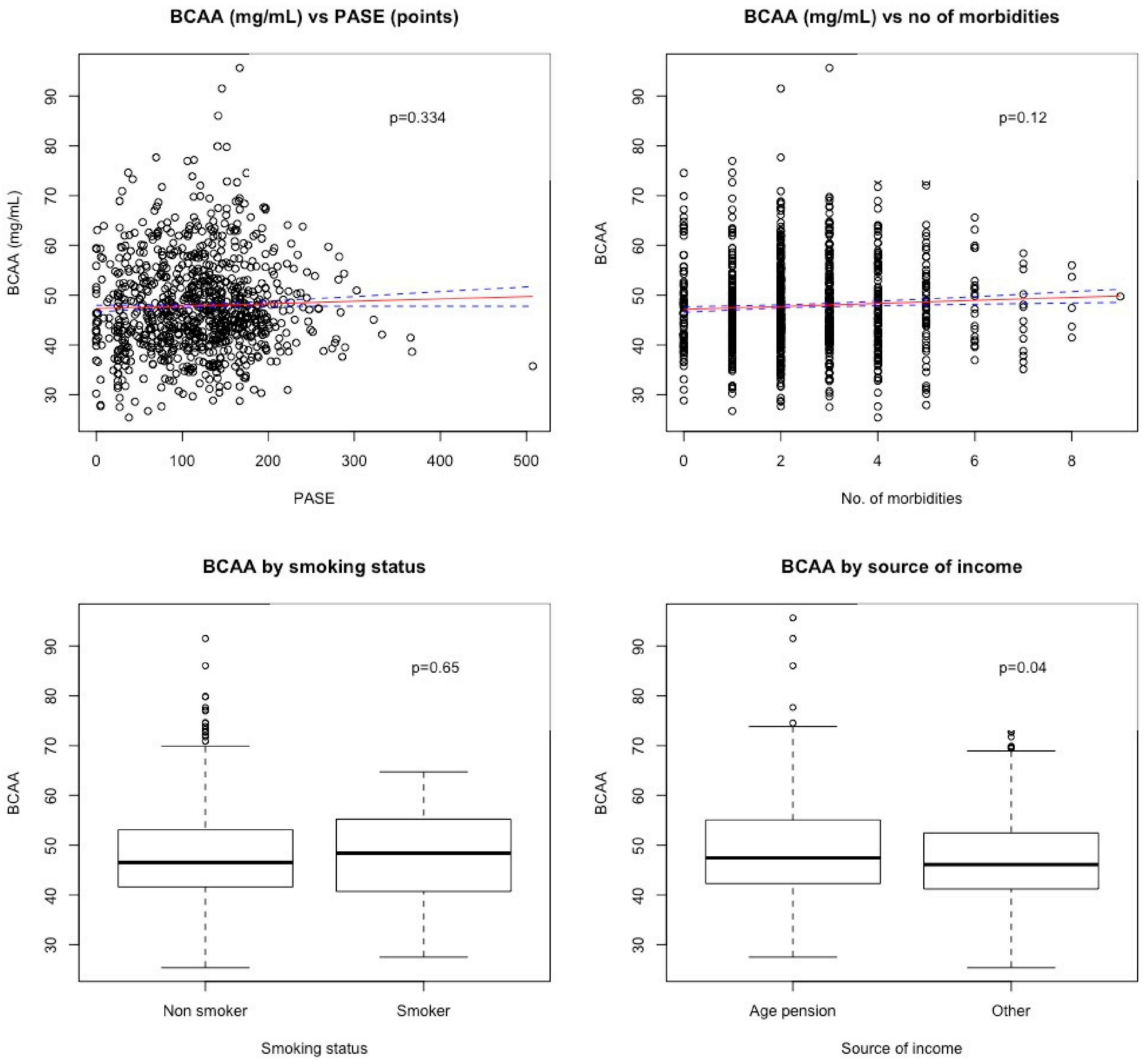

BCAA, branched-chain amino acid; PASE, Physical Activity Scale for the Elderly

Circulating branched-chain amino acids and their association with age, physical activity level (as measured by Physical Activity Scale for the Elderly), number of morbidities, income and smoking status in the human cohort.
